# Selective Removal of Iron, Lead, and Copper Metal Ions from Industrial Wastewater by a Novel Cross-Linked Carbazole-Piperazine Copolymer

**DOI:** 10.3390/polym14122486

**Published:** 2022-06-18

**Authors:** Majed Al Anazi, Ismail Abdulazeez, Othman Charles S. Al Hamouz

**Affiliations:** 1 Chemistry Department, King Fahd University of Petroleum and Minerals, Dhahran 31261, Saudi Arabia; alanazimajed1@gmail.com; 2 Interdisciplinary Research Center for Membranes and Water Security, King Fahd University of Petroleum and Minerals, Dhahran 31261, Saudi Arabia; ismail.abdulazeez@kfupm.edu.sa; 3 Interdisciplinary Research Center for Hydrogen and Energy Storage, King Fahd University of Petroleum and Minerals, Dhahran 31261, Saudi Arabia

**Keywords:** polyamine, industrial wastewater treatment, heavy metal ions

## Abstract

A novel cross-linked Copolymer (***MXM***) was synthesized by the polycondensation reaction of 3,6-Diaminocarbazole and piperazine with *p*-formaldehyde as a cross-linker. The Copolymer was fully characterized by solid ^13^C-NMR and FT-IR. The thermal stability of ***MXM*** was investigated by TGA and showed that the Copolymer was stable up to 300 °C. The synthesized polyamine was tested for the removal of iron (*Fe*^2+^), lead (*Pb*^2+^), and copper (*Cu*^2+^) ions from aqueous and industrial wastewater solutions. The effect of pH, concentration and time on the adsorption of iron (*Fe*^2+^), lead (*Pb*^2+^), and copper (*Cu*^2+^) ions was investigated. The adsorption of the studied ions from aqueous solutions onto the ***MXM*** polymer occurs following the Freundlich isotherm and pseudo-second-order kinetic models. The intraparticle diffusion model showed that the adsorption mechanism is controlled by film diffusion. The regeneration of ***MXM*** showed practical reusability with a loss in capacity of 2–5% in the case of *Fe*^2+^ and *Cu*^2+^ ions. The molecular simulation investigations revealed similarities between experimental and theoretical calculations. Industrial wastewater treatment revealed the excellent capabilities and design of ***MXM*** to be a potential adsorbent for the removal of heavy metal ions.

## 1. Introduction

The growth of population and industrialization has resulted in a massive quantity of hazardous chemicals being released into the environment, posing a warning to human life and the quality of the urban environment [1]. Due to their apparent cytotoxicity, even at low quantity levels, heavy metal pollution has been a major and serious problem globally in the last several decades. As a result, it is critical to continually monitor heavy metal pollution and health risks in cities, particularly mining and industrial areas [2].

Although iron is not harmful to one’s health, it is regarded as a secondary or cosmetic contaminant. An increase in iron concentration in the human body could cause health issues, although it is necessary for health at a certain level since it aids in transferring oxygen in the blood [3]. However, iron contamination in the oil industry can be devastating as iron poisoning affects fluid catalytic-cracking catalysts. After iron contamination, a thick coating can build on the catalyst’s surface, which prevents reactants from diffusing into the catalyst’s inner structures. This poisoning has a significant detrimental impact on the catalyst’s capacity to convert heavy oils, and it might significantly reduce gasoline output while significantly boosting dry gas, coke, and slurry yields [4,5].

On the other hand, copper becomes poisonous in high quantities and can damage the brain, heart, and kidneys of human beings [6]. Moreover, it can disrupt the metabolic activities of marine species’ where copper concentrations greater than 0.08 µM, for example, have a deleterious effect on the completion of several life periods in brown macroalgae [7]. In the industry, the presence of copper ions can decrease the selectivity of extracting nickel and iron in the mineral processing industry [8].

While pollution with lead is considered a serious and avoidable environmental health issue for children, infants are more vulnerable to lead exposure than adults [9]. Multiple organ systems are affected by lead exposure, resulting in significant morphological, biochemical, and physiological alterations. Chronic lead poisoning has been linked to fatigue, sleep difficulties, headaches, stupor, and anemia [10]. In the industry, catalysts that contain noble metals such as Rhodium (Rh), Palladium (Pd), and Platinum (Pt) in their structure are sensitive to lead [11].

Many techniques for water/wastewater treatment have been developed and used by scientists; adsorption, evaporation, reverse osmosis, filtration, electrolysis, flocculation, sedimentation/gravity extraction, screening, precipitating, oxidation, coagulation, solvent extraction, distillation, solidification, ion exchange, and centrifugation are some of the techniques used [12,13]. However, adsorption remains a preferable approach to other techniques due to the simplicity of obtaining a large field of adsorbents that are significantly valuable, cost-effective, ecologically acceptable, and simple to use. However, effective adsorption is dependent on the use of an effective adsorbent, necessitating ongoing studies in this area [14,15,16].

Polymers are an important type of adsorbent that have recently captured significant attention in the removal of heavy metal ions from wastewater solutions. Polymers have shown resilience and selectivity in removing heavy metals from aqueous solutions due to their effectiveness, durability, variability, ease of design, and low cost. Such polymers are carbazole containing polymers that have been used to remove heavy metal ions. Recently, carbazole-containing polymers have been used as sensors for detecting mercury (II) ions in aqueous solutions [17]. Hypercrosslinked microporous carbazole-based polymer has been investigated for the removal of lead ions with a % removal of 99.8% [18]; another carbazole based porous organic framework (CzBPOF) has also been used for the removal of lead (II) ions from water with a % removal of 92.56% within 80 min [19].

Most reported carbazole-based polymers have never been tested on industrial wastewater treatment under actual conditions. In our endeavor in the design and application of polymers for the treatment of heavy metal ions, a novel cross-linked Copolymer containing diaminocarbazole and piperazine has been synthesized as an effective adsorbent for the removal of iron (*Fe*^2+^), lead (*Pb*^2+^), and copper (*Cu*^2+^) ions from wastewater solutions.

## 2. Experimental

### 2.1. Materials and Equipment

3,6-Diaminocarbazole, piperazine, *p*-formaldehyde, glacial acetic acid, methanol, and DMF were purchased from Sigma Aldrich and were used without further purification. Metal adsorption analysis was performed using ICP-OES Optima 8000 Perkin Elmer. Polymer structure was determined by ^13^C-NMR spectra using solid-state type and was recorded by Bruker Avance III—400 WB. FTIR spectra were produced on iTR Nicolet is 10 spectrometer. pH measurements were performed using HACH HQ411D. Thermal stability was measured using (TGA) Q600 TA instruments at a heating rate of 20 °C/min under a nitrogen atmosphere.

### 2.2. Polymer Synthesis (**MXM**)

In a typical reaction [20], 3,6-Diaminocarbazole (0.01 mmol, 1.97 g), piperazine (0.04 mmol, 3.44 g), and *p*-formaldehyde (0.04 mmol, 1.201 g) in DMF (25 mL) were stirred for 10 min and in a 100 mL PTFE liner hydrothermal autoclave vessel. The vessel was purged with nitrogen and sealed. The reaction vessel was transferred into a stainless-steel reaction vessel and heated in an oven at 100 °C for 24 h. Once completed, the reaction mixture was filtered and washed with methanol. The dark black solid powder was then dried under vacuum at 70 °C for 24 h (Yield: 5.1 g, 77%).

### 2.3. Adsorption Experiments

Batch adsorption studies were performed on the capability of ***MXM*** to remove three metal ions *Fe*^2+^, *Cu*^2+^, and *Pb*^2+^. In a typical experiment [21], 30 mg of the polymer was added to a 20 mL metal solution at a certain pH, initial concentration, and temperature and stirred for a specific amount of time. Once the adsorption experiment was completed, the reaction mixture was filtered, and the concentration of the metal ions after adsorption was measured. The capability of ***MXM*** was found by calculating the adsorption capacity as described in the following equation:

(1) $$Q_{e}=\frac{\left(C_{o}-C_{f}\right)\times V}{W}$$

where $Q_{e}$ is the adsorption capacity (mg/g), and *C_o_* and *C*_f_ are the initial and final concentrations of metal ions (mg/L) in the solution, respectively. *W* is the mass of ***MXM*** (mg), and V is the volume of the metal ion solution (L).

### 2.4. Molecular Simulation

Molecular simulations were conducted using the COMPASS II forcefield [22] on Materials Studio 8.0 suite to determine the density and the fractional free volume (FFV) of the material, ***MXM***. An amorphous cell comprising 20 units of ***MXM*** was constructed and minimized using the Forcite module, followed by dynamics simulations on the NPT and the NVT ensembles each for 1000 ps at a time step of 1.0 fs. The Nose–Hoover thermostat and the Berendsen barostat were used to control the temperature (at 298 K) and pressure (at 8 bar), respectively. The Ewald summation method was used for long-range Coulombic interactions, while Lennard-Jones interactions were estimated with a cut-off range of 12.5 Å. The interaction of ***MXM*** and metal ions were simulated by conducting quantum mechanical DFT simulations on Gaussian 09 [23], at the B3LYP exchange-correlation functional, in combination with the Pople’s split valence basis set, 6-311G*, and the SDD pseudopotential basis set, for the non-metal and metal atoms, respectively [24,25]. The polarized continuum model–self-consistent reaction field (PCM-SCRF) model was used to simulate the aqueous media, with the solvent depicted as water [26]. After full geometry optimization, the adsorption energies of ***MXM*** and the metal ions were computed using the following equation:

(2) $$E_{ad}=E_{M-MXM}-E_{M}-E_{MXM}$$

where *E_M-**MXM**_* represents the total energies of the metal ion-***MXM*** complexes, while *E_M_* and *E**_MXM_*** represent the isolated energies of the metal ions and ***MXM***, respectively. The nature of the interactions was explored using the reduced density gradient (RDG) isosurfaces plots as implemented on the multi-wavefunction analysis code [27].

## 3. Results and Discussion

**Scheme 1:**
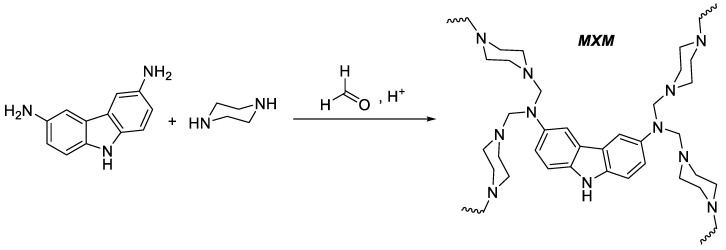
Synthesis of ***MXM*** Copolymer.

A novel cross-linked Copolymer has been synthesized by the polycondensation reaction of 3,6-Diaminocarbazole and piperazine with *p*-formaldehyde to produce the ***MXM*** Copolymer (Scheme 1). The monomeric moieties of diaminocarbazole and piperazine were chosen to be typical for an adsorbent of heavy metal ions as they provide the rigidity of aromatic moieties in diaminocarbazole and the flexibility of piperazine units. The high amount of nitrogen atoms present in these monomers provides a high concentration of attractive adsorption sites for metal ions to be attached and removed.

### 3.1. MXM Copolymer Structure Characterization

**Figure 1:**
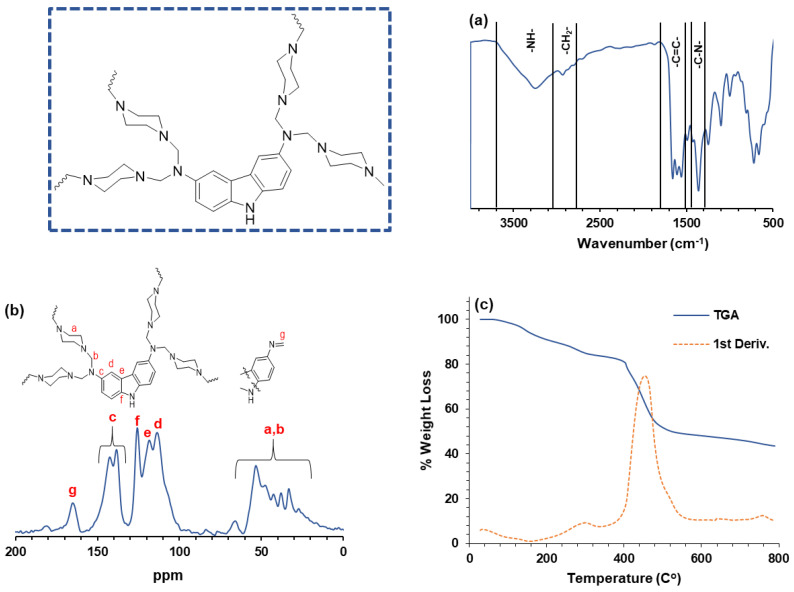
(**a**) FT−IR spectrum of ***MXM*** Copolymer. (**b**) The solid ^13^C−NMR spectrum of ***MXM*** Copolymer. (**c**) Thermogravimetric analysis (TGA) and the first-derivative of ***MXM*** Copolymer.

Copolymer ***MXM*** was characterized by solid ^13^C-NMR and FT-IR, as shown in Figure 1a,b. The Figure elucidates the characteristic features of the Copolymer. The FTIR spectrum in Figure 1a shows an absorption band at ~3400 cm^−1^, which represents the –N–H stretching frequency of ***MXM***, and an absorption band at ~2980 cm^−1^, which represents the –CH_2_– stretching vibration of the methylene units in piperazine moiety, and the methylene bridge that links the diaminocarbazole with the piperazine monomers. An absorption band at ~1600 cm^−1^ represents the –C=C– stretching vibrations of the benzene rings found in the diaminocarbazole monomer. An absorption band at ~1440 cm^−1^ represents the –C–N– stretching vibration in both monomeric units [28,29]. Figure 1b represents the solid ^13^C-NMR spectrum for ***MXM***; the spectrum shows multiple peaks ~30–50 ppm, referred to as the methylene units in the piperazine monomer and the methylene bridge between the diaminocarbazole and piperazine monomer. The peaks of ~100–150 ppm are characteristic of the aromatic carbons found in diaminocarbazole [30,31,32]. The peak present ~165 ppm is due to the formation of imine linkage between terminal amines with *p*-formaldehyde. Figure 1c shows the thermogravimetric analysis of the ***MXM*** Copolymer. The thermogram shows two degradation steps; the first thermal degradation step at ~300 °C corresponds to the thermal degradation of aliphatic chains in the piperazine units and methylene bridges between monomeric units, followed by a sharp thermal degradation at ~500 °C due to the carbonization of diaminocarbazole aromatic units [33,34].

### 3.2. MXM Copolymer Adsorption Properties

To fully comprehend the capability of the ***MXM*** Copolymer to adsorb heavy metal ions, the Copolymer was studied under different adsorption conditions experimentally. Moreover, the Copolymer was studied under molecular simulation to study the affinity of the ***MXM*** Copolymer toward the adsorption of lead, iron, and copper metal ions.

#### 3.2.1. Effect of pH on the Adsorption Capacity of ***MXM***

**Figure 2:**
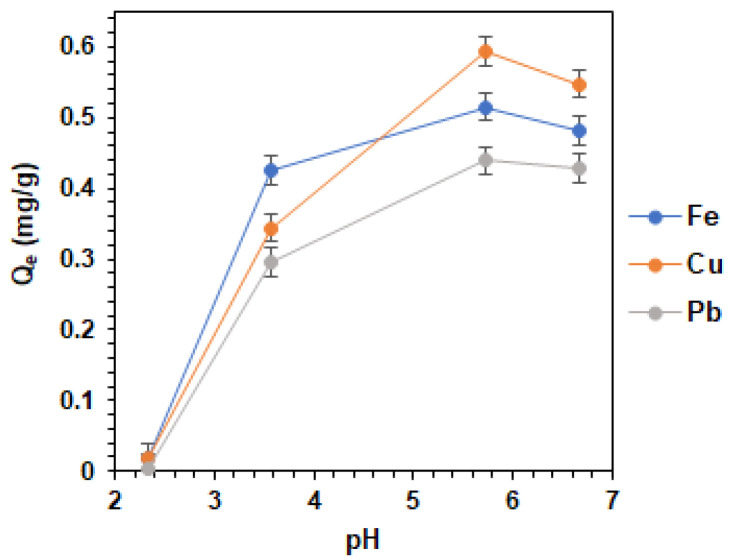
The effect of pH on the adsorption capacity of the ***MXM*** Copolymer.

**Scheme 2:**
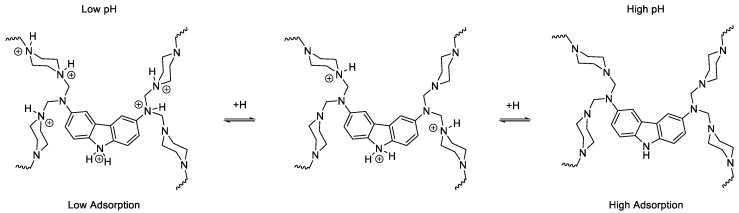
Effect of pH on the protonation of ***MXM*** the Copolymer.

The synthesized Copolymer ***MXM*** was tested at different pHs (2.34, 3.57, 5.73, and 6.67) to find out the adsorption efficiency of ***MXM*** on a 1 ppm solution of iron (*Fe*^2+^), copper (*Cu*^2+^), and lead (*Pb*^2+^) ions. In a typical experiment, 30 mg of ***MXM*** was added to a 20 mL metal ion and stirred for 1 h. Once completed, the solution was filtered, and the concentration of metal ions was measured. Figure 2 depicts the influence of pH on the adsorption capacity of ***MXM***. As shown in the figure, the adsorption capacity of ***MXM*** increases as pH increases. In high acidic conditions, the increased protonation of the adsorbent surface results in lower metal ion adsorption, as shown in Scheme 2. The pH also has an impact on the speciation of heavy metal ions. The adsorption experiments were performed in the pH range of 2–6; after pH = 6, the metal ions start precipitating, which is expected due to the formation of metal hydroxides M(OH)_2_. As a result, pH = 5.73 was utilized for the rest of the adsorption studies.

#### 3.2.2. Effect of Initial Metal Ion Concentration on the Adsorption Capacity of ***MXM*** Copolymer

**Figure 3:**
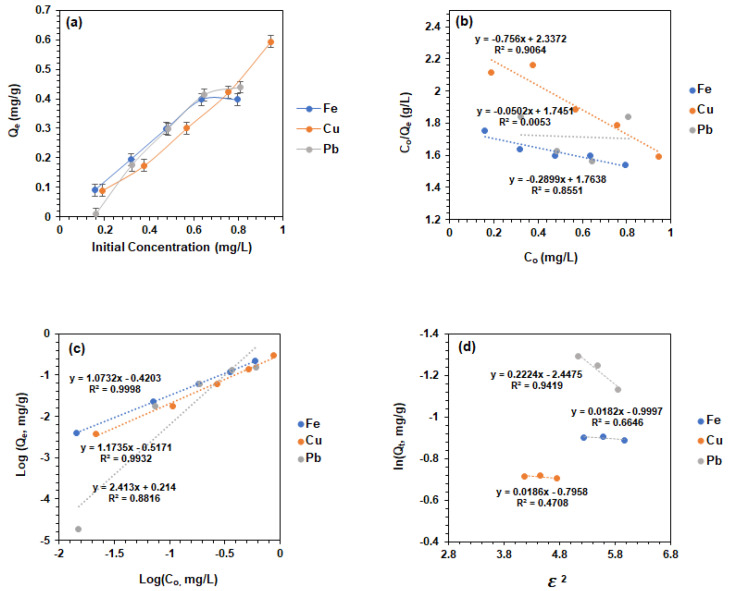
(**a**) Effect of metal ion initial concentration on the adsorption capacity of ***MXM***; (**b**) Langmuir isotherm model for the adsorption of iron(II), Cu(II), and lead(II) ions by ***MXM***; (**c**) Freundlich isotherm model for the adsorption of iron(II), Cu(II), and lead(II) ions by ***MXM***; (**d**) DKR isotherm model for the adsorption of iron(II), Cu(II), and lead(II) ions by ***MXM***.

Figure 3a demonstrates the influence of the initial metal ion concentration (mg/L) on the adsorption capacity of ***MXM***. The adsorption investigation was carried out on three metal ion solutions (*Cu*^2+^, *Fe*^2+^, and *Pb*^2+^) with a concentration ranging from 0.2 to 1 (mg/L) at pH = 5.73 and a specific time of 1 h. The figure shows that the adsorption capacity increases with increases in the initial concentration of the metal ion. Three adsorption isotherm models were used to analyze the experimental data acquired by the batch adsorption studies; Langmuir, Freundlich, and the Dubinin–Kaganer–Radushkevich (DKR) isotherm model. The Langmuir isotherm model describes adsorption as a single-layer adsorption where one metal is attached to one adsorption site (Figure 3b). It depicts the uniform adsorption of metal ions on the surface of the adsorbent [35]. The negative values shown in Table 1 reveal that the adsorption process of the three metal ions does not follow the Langmuir isotherm model. In contrast to the Langmuir isotherm, the Freundlich isotherm model explains the adsorption behavior when the surface is heterogeneous, where one active site can accommodate more than one metal ion [36,37]. The heterogeneity of the surface can be determined from the slope (1/*n*). High heterogeneity is described when the value of 1/*n* is ~0. When the value of 1/*n* < 1, adsorption is considered favorable. If the slope (1/*n*) has a value above one, this indicates cooperative adsorption. As shown in Table 1 (Figure 3c), the values of 1/*n* are higher than 1, indicating that the adsorption process is cooperative in nature [38]. The DKR model [39], which is applied to single solute systems, describes the adsorption mechanism as physical or chemical in nature [40]. The results shown in Table 1 (Figure 3d) show that adsorption energy E is exothermic, indicating favorable adsorption [40].

#### 3.2.3. The Effect of Time on the Adsorption Capacity of ***MXM***

**Figure 4:**
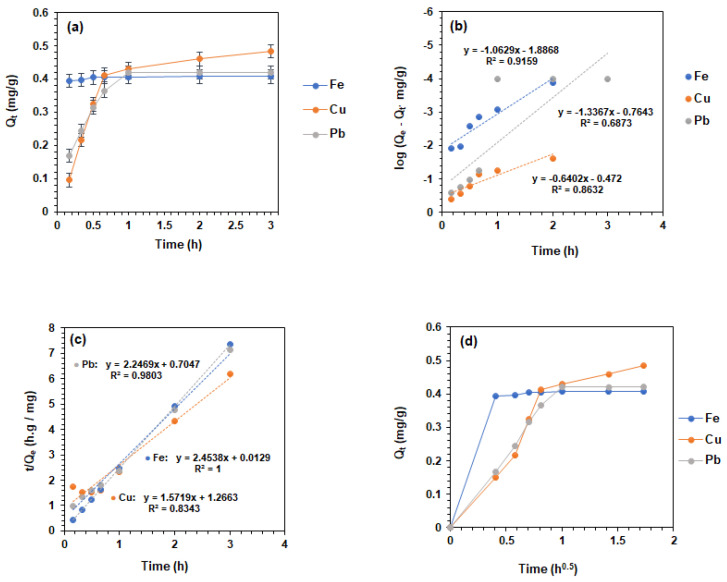
(**a**) The effect of time on the adsorption capacity *Fe*^2+^, *Cu*^2+^, and *Pb*^2+^ by ***MXM***. (**b**) PFO kinetic model on the adsorption capacity *Fe*^2+^, *Cu*^2+^, and *Pb*^2+^ by ***MXM***. (**c**) PSO kinetic model on the adsorption capacity *Fe*^2+^, *Cu*^2+^, and *Pb*^2+^ by ***MXM***. (**d**) Intraparticle diffusion model on the adsorption capacity *Fe*^2+^, *Cu*^2+^, and *Pb*^2+^ by ***MXM***.

The adsorption rate, which determines how long it takes for the adsorption process to reach equilibrium, is also influenced by adsorption kinetics and is shown in Figure 4. This information is critical for the process of innovation and adsorption system design. In this work, three kinetic models were used to analyze experimental data: pseudo first-order (PFO), pseudo second-order (PSO), and intraparticle diffusion (IPD), which were used to define the adsorption kinetics of *Fe*^2+^, *Cu*^2+^, and *Pb*^2+^ by the ***MXM*** Copolymer.

PFO kinetic model posits that the rate of change of adsorbed solute by time is precisely comparative to the change in saturation concentration and the quantity of solid adsorption with time, and it may be applied to any adsorption process in the first stage [41,42] from the data shown in Table 2 (Figure 4b). The *Q*_e(exp)_ values are not close to the values of *Q_e_* found by PFO, which implies that the experimental data does not fit the PFO kinetic model. PSO’s premise is that the rate-limiting phase is chemisorption, and it predicts the behavior over the entire adsorption range [43,44]. In this situation, the adsorption rate is governed by the adsorption capacity rather than the adsorbate concentration. The model can determine the equilibrium adsorption, which is a key advantage of PSO over PFO; hence, no requirement is needed to assess the adsorption equilibrium capacity from the experiment.

As shown in Table 2 (Figure 4c), *Q*_e(exp)_ is close to the *Q_e_* values calculated in the PSO model with good regression values, implying that the adsorption process is chemisorption [45,46].

Weber and Morris devised an intraparticle diffusion model to determine the adsorption diffusion process. Nonlinearity, on the other hand, is occasionally found. When the data show several linear plots, it implies that adsorption was caused by more than one mechanism. As a result, these rate-determining processes may be split into multiple linear curve segments throughout time, each having control over the entire process [47,48]. The discovery of the rate-limiting step provides new insights into the mechanism of the adsorption process. According to the intraparticle diffusion model, first, metal ions move interfacially (i.e., film diffusion) between the absorbent and solution. Second, a rate-limiting intraparticle diffusion phase carries the ions into the adsorbent sites, and then adsorption reaches equilibrium [49]. From Figure 4d, the adsorption of *Fe*^2+^, *Cu*^2+^, and *Pb*^2+^ metal ions by Copolymer ***MXM*** proceeds through two steps, with film diffusion followed by equilibrium.

### 3.3. Reusability of **MXM**

**Figure 5:**
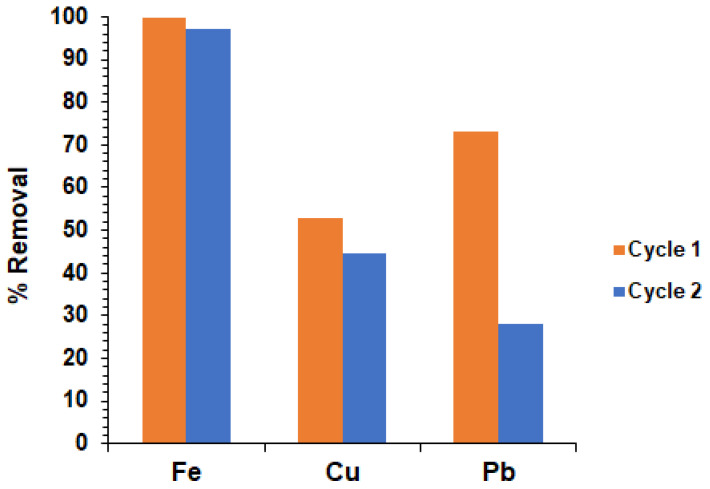
Regeneration capability of ***MXM*** polymer.

The reusability of ***MXM*** is a vital factor for its use in industrial wastewater treatment; for that, ***MXM*** was subjected to two cycles of adsorption, as shown in Figure 5. In a typical experiment, 100 mg of ***MXM*** was placed in a 20 mL 1 ppm solution of *Pb*^2+^, *Fe*^2+^, and *Cu*^2+^ for 1 h under stirring. Once the experiment was completed, the adsorption capacity was measured. The polymer was soaked in a 1M solution of nitric acid for 15 min, followed by washing with water and 1M sodium hydroxide; then, it was thoroughly washed with water. The experiment was repeated, and the adsorption capacity of ***MXM*** was measured again. The adsorption experiments reveal the usability of ***MXM*** with small loss of activity in the adsorption of *Fe*^2+^ and *Cu*^2+^, which proves the suitability of ***MXM*** to be used as an adsorbent.

### 3.4. Molecular Simulation

**Figure 6:**
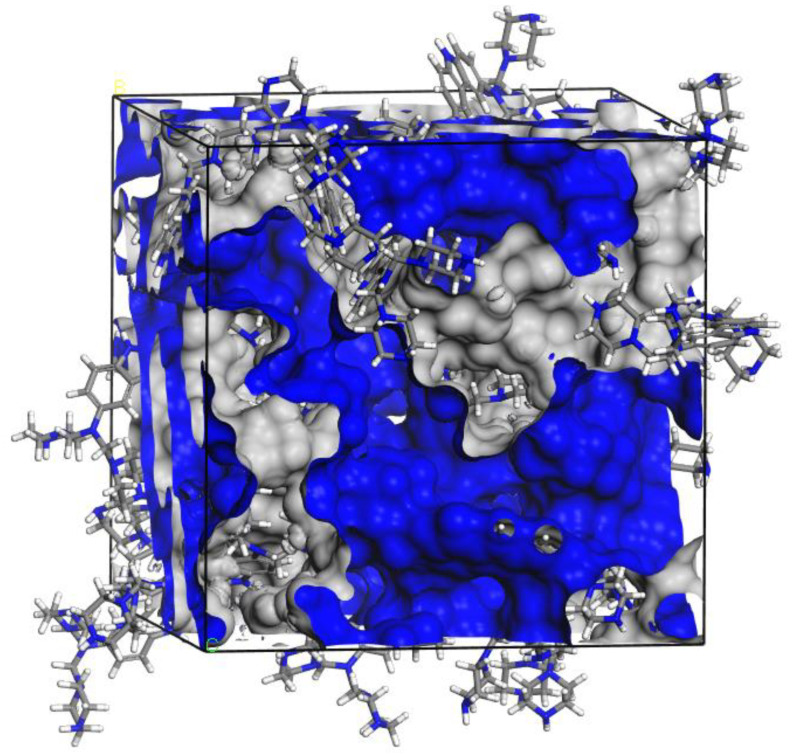
Energy-minimized amorphous cell comprising ***MXM***.

The results of MD simulations on 20 units of ***MXM*** are shown in Figure 6. The grey and blue isosurfaces represent the accessible free volume on ***MXM*** at a probe radius of 1.09 Å. The system was stabilized mainly by intermolecular hydrogen bonds and possible π-π stacking between the aromatic rings. The estimated FFV of ***MXM*** (0.15) indicates moderate hydrogen bonding within the system and suggests higher water permeance and greater mobility of the metal ions in good correlation with fully reported aromatic systems [50].

**Figure 7:**
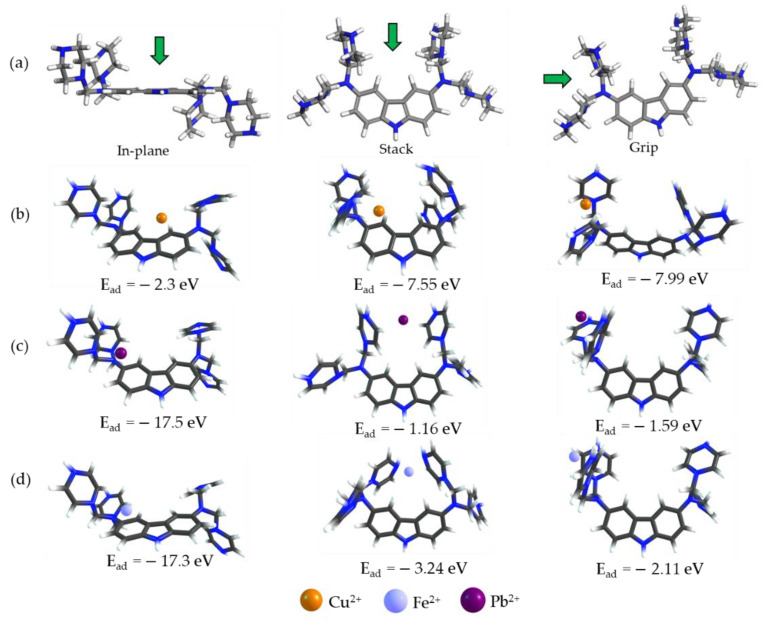
Molecular level adsorption of *Cu*^2+^, *Pb*^2+^, and *Fe*^2+^ ions on ***MXM*** at the B3LYP/6-311G* and SDD levels of theory. (**a**) The optimized adsorption configurations of the metal ions on ***MXM***, and the molecular level adsorption of (**b**) *Cu*^2+^, (**c**) *Pb*^2+^ and (**d**) *Fe*^2+^ ions at the B3LYP/6-311G* and SDD levels of theory.

Molecular level quantum simulations of the adsorption of *Cu*^2+^, *Pb*^2+^, and *Fe*^2+^ ions on ***MXM*** molecules were conducted and presented in Figure 7. Three possible adsorption modes, the in-plane, the stack, and grip configurations, were identified and geometrically optimized. The adsorption energies, E_ad_, revealed that ***MXM*** exhibited higher adsorption capacity toward *Cu*^2+^ ions with energies of −22.3, −7.55, and −7.99 eV for the in-plane, the stack, and the grip configurations, respectively. The order of E_ad_, *Cu*^2+^ > *Pb*^2+^ > *Fe*^2+^, was consistent with the change in the frontier molecular orbital (FMO) energy separation of ***MXM*** upon the adsorption of the metal ions (Figure S1), which revealed the stabilization of the nucleophilic character of the Copolymer material by lowering the energy of the highest occupied molecular orbital. Consequently, a significant fraction of charges was transferred to the Copolymer materials when interacting with *Cu*^2+^ ions, resulting in higher adsorption capacity that is consistent with the experimental data.

The RDG isosurface plots of the various optimized adsorption modes of the metal ions on the active sites of ***MXM*** are presented in Figure S2. It mainly depicts non-covalent interactions (NCIs) characterized by electron density ρ [51,52]. RDG isosurface plots revealed predominantly weak Van der Waals interactions between ***MXM*** and the metal ions. In-plane geometry exhibited higher ρ along the plane of interactions, suggesting stronger adsorption as revealed by E_ad_, and this is consistent with the change in FMO distribution. Meanwhile, partial repulsions were exhibited by *Pb*^2+^ and *Fe*^2+^ ions, resulting in slightly weaker adsorptions, in good correlation with the experimental adsorption data.

### 3.5. Wastewater Sample Treatment

The significant element in synthesizing an adsorbent polymer is to find out its ability to treat wastewater. The effectiveness of ***MXM*** in removing heavy metals was tested using two samples of wastewater (unspiked, spiked each by 1 mg/L iron, copper, and lead ions). The wastewater sample had a pH of 7.9, total dissolved solids of 1096.4 mg/L, total suspended solids of 11 mg/L, chlorides of 254 mg/L, and organic carbon of 9.8 mg/L. A 20 mL sample of unspiked and spiked samples containing the three metal ions was treated with ***MXM***. The results in Table 3 reveal the efficiency of ***MXM*** as an adsorbent for removing *Fe*^2+^, *Cu*^2+^, and *Pb*^2+^ from wastewater solutions. A comparison Table between ***MXM*** and other adsorbents reported in the literature shows the efficiency of ***MXM*** as an adsorbent for the removal of heavy metal ions (Table S1).

## 4. Conclusions

A novel cross-linked carbazole-piperazine Copolymer has been synthesized, and the adsorption properties were investigated under different conditions such as pH, initial metal ion concentration, and time. The adsorption results revealed that the ***MXM*** polymer followed the Freundlich isotherm model, where the adsorption process is heterogeneous, whereas the adsorption kinetics followed the pseudo-second-order kinetic model indicating chemisorption. The intraparticle diffusion model indicated that adsorption experiences film diffusion followed by equilibrium. The regeneration of ***MXM*** polymer showed its ability to be used again with little losses of 2–5% in capacity in the case of iron and copper, which makes it effective as an adsorbent. Interestingly, the performance of ***MXM*** was the most efficient in the case of industrial wastewater treatment, where ***MXM*** showed higher adsorption capacity toward iron and lead than copper, which could be due to the different environments, pH, and salinity of the industrial wastewater sample with a % removal of 55–100% removal capacity. The results indicate the strong potential of ***MXM*** to be an efficient adsorbent for the removal of heavy metal ions from wastewater solutions.

## Figures and Tables

**Table 1:** The isothermal constants of the three models Langmuir, Freundlich, and DKR.

Metal Ion		*Langmuir Isotherm Model*		
		*Q_m_*	*b*	R^2^
*Fe* ^2+^		−3.45	−0.164	0.8551
*Cu* ^2+^		−1.32	−0.323	0.9064
*Pb* ^2+^		−19.9	−0.029	0.0053
		***Freundlich isotherm model***		
		*k_f_*	1/*n*	R^2^
*Fe* ^2+^		0.380	1.07	0.9998
*Cu* ^2+^		1.17	0.304	0.9932
*Pb* ^2+^		2.41	1.64	0.8816
	***DKR isotherm model***			
	*Q_m_*	β	E	R^2^
*Fe* ^2+^	1.02	0.999	−0.707	0.6646
*Cu* ^2+^	1.02	0.796	−0.793	0.4708
*Pb* ^2+^	1.25	2.44	−0.452	0.9419

**Table 2:** PFO and PSO kinetic model constants for the adsorption of the *Fe*^2+^, *Cu*^2+^, and *Pb*^2+^ by ***MXM***.

Metal Ion		*PFO Kinetic Model*		
	*Q_e_* exp	*q_e_*	*k*_1_	R^2^
*Fe* ^2+^	0.407	0.087	4.345	0.9159
*Cu* ^2+^	0.484	0.228	1.087	0.8632
*Pb* ^2+^	0.420	0.046	3.078	0.6873
		***PSO Kinetic Model***		
	*Q_e_* exp	*q_e_*	*k*_2_	R^2^
*Fe* ^2+^	0.407	0.408	466.8	1.000
*Cu* ^2+^	0.484	0.636	1.952	0.8343
*Pb* ^2+^	0.420	0.445	7.165	0.9803

**Table 3:** Comparison of the metal ions concentrations before and after treatment of wastewater sample (unspiked and spiked) and spiked DI water.

Metal Ions	*Unspiked Wastewater Sample*		
	Original sample	After Treatment	% Removal
*Fe* ^2+^	0.061	0.00	100
*Cu* ^2+^	0.003	0.002	33.3
*Pb* ^2+^	-	-	-
	***Spiked Wastewater Sample***		
	Original sample	After Treatment	% Removal
*Fe* ^2+^	0.454	0	100
*Cu* ^2+^	0.485	0.219	54.8
*Pb* ^2+^	0.376	0.015	96

## Data Availability

Data presented in this study are available upon fair request to the corresponding author.

## Supplementary Materials

The following supporting information can be downloaded at: https://www.mdpi.com/article/10.3390/polym14122486/s1, Figure S1: The change in frontier molecular orbital distribution of (a) ***MXM*** when interacting with (b) *Cu*^2+^, (c) *Pb*^2+^, and (d) *Fe*^2+^ ions at the B3LYP/6-311G* and SDD levels of theory; Figure S2: The reduced density gradient (RDG) isosurface plots of the interactions of ***MXM*** with (a) *Cu*^2+^, (b) *Pb*^2+^, and (c) *Fe*^2+^ ions; Table S1: Comparison between ***MXM*** and adsorbents for the removal of heavy metal ions [53,54,55,56,57,58,59].
