# Supplementary material for: Selective Removal of Iron, Lead, and Copper Metal Ions from Industrial Wastewater by a Novel Cross-Linked Carbazole-Piperazine Copolymer
Source: Polymers (Basel). 2022 Jun 18;14(12):2486. doi: 10.3390/polym14122486 (PMC9227031; doi:10.3390/polym14122486)
Supplement: Supplementary file 1 [file polymers-14-02486-s001.zip › polymers-1756623-supplementary.pdf]

Selective Removal of Iron, Lead, and Copper Metal ions from Industrial Wastewater by a Novel Cross-linked Carbazole-Piperazine Polymer

Majed Al Anazi[1], Ismael abdulaziz[2], Othman Charles S. Al Hamouz*[1,3]

[1]Chemistry Department, King Fahd University of Petroleum and Minerals, Dhahran, 31261, Saudi Arabia.
[2]Interdisciplinary Research Center for membranes and water security, King Fahd University of Petroleum and Minerals, Dhahran, 31261, Saudi Arabia.
[3]Interdisciplinary Research Center for Hydrogen and Energy Storage, King Fahd University of Petroleum and Minerals, Dhahran, 31261, Saudi Arabia.
Corresponding Author E-mail: othmanc@kfupm.edu.sa; (Cell: +966552709130)

1. Molecular Simulation

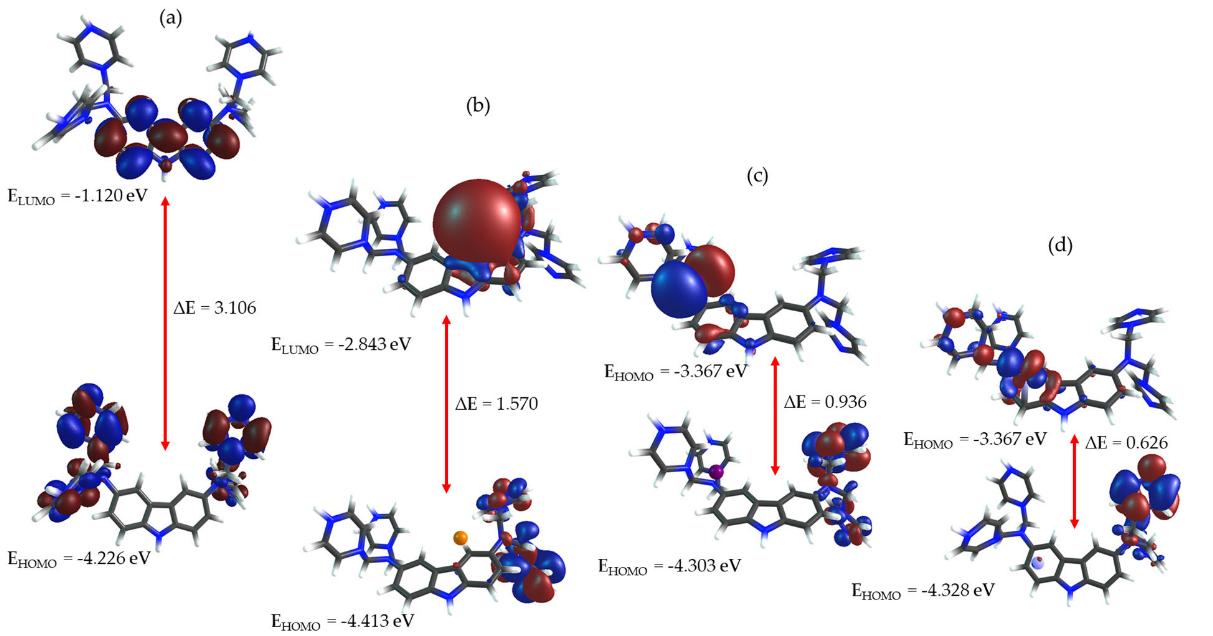

Figure S1. The change in frontier molecular orbital distribution of (a) ***MXM*** when interacting with (b) Cu^{2+}, (c) Pb^{2+} and (d) Fe^{2+} ions at the B3LYP/6-311G* & SDD levels of theory.

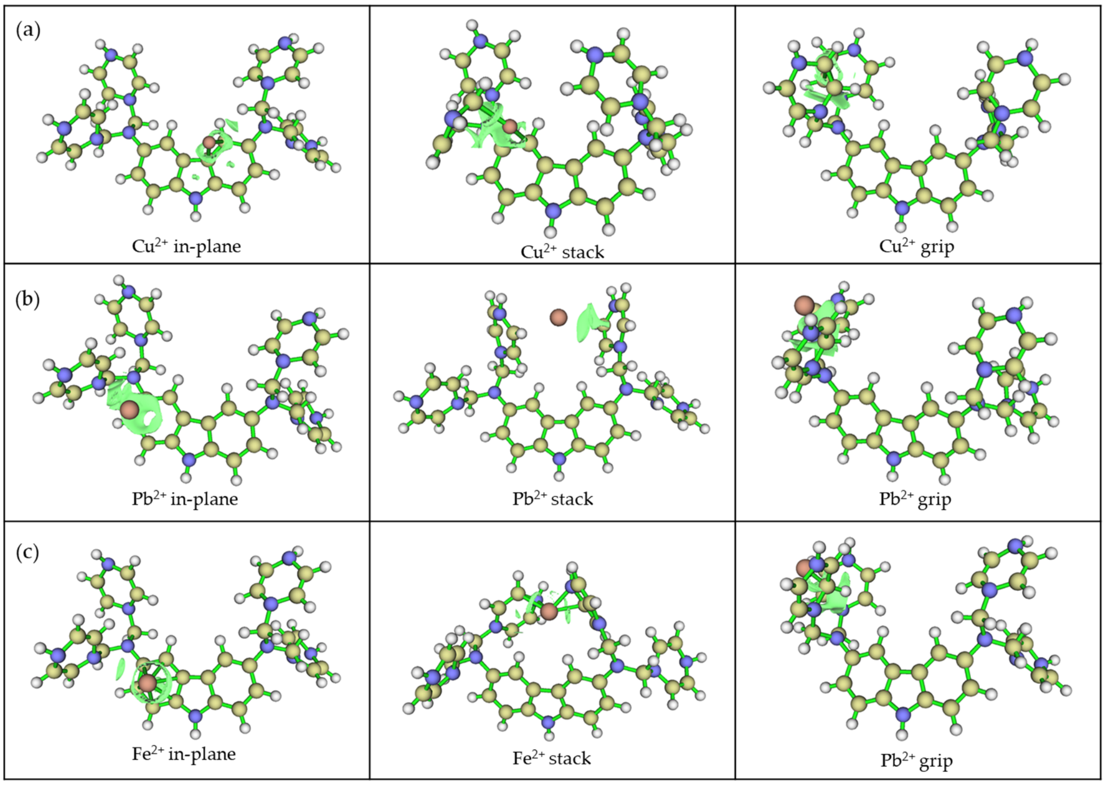

Figure S2. The reduced density gradient (RDG) isosurface plots of the interactions of ***MXM*** with (a) Cu^{2+}, (b) Pb^{2+} and (c) Fe^{2+} ions

2. Swelling properties

In a typical experiment; a 0.1 g of MXM polymer was weighed before the swelling experiment and was recordered as M_o. the sample was immersed in distill water for 24 hours. once the time was elapsed the sample surface was dried. The sample was weighed again (M_f) and the % swelling was measured as follow:

$$\%\ Swelling = \frac{M_f - M_o}{M_o} * 100 \qquad (1)$$

The results reveled that the % swelling was found 15 %.

3. Comparison table with literature:

Table S1. Comparison between ***MXM*** and adsorbents for the removal of heavy metal ions.

	% Removal			
Material	Fe	Pb	Cu	Reference
Carbon nanotube/G composite	-	-	83.3	[1]
Titanate nanotube/G nanocomposite	-	-	98	[2]
Chitosan	99.3	-	90	[3]
PE/PP non-woven fabric grafted with poly(bis[2-(methacryloyloxy) ethyl] phosphate)	-	-	60	[4]
γ-polyglutamic acid (γ-PGA) functionalized lignin (DLGS)	-	99.24	56	[5]
and ε-poly-L-lysine (ε-PL) functionalized lignin (DLLS)	-	99.04*	54*	[5]
poly (hydroxamic acid) ligand	99.3	-	90	[6]
phytic acid-functionalized spherical poly-phenylglycine	-	95	91	[7]
MXM	100*	96*	55*	This work

* Mixed metal ion solution

References

1. Li, Y.; Liu, F.; Xia, B.; Du, Q.; Zhang, P.; Wang, D.; Wang, Z.; Xia, Y., Removal of copper from aqueous solution by carbon nanotube/calcium alginate composites. *Journal of Hazardous Materials* **2010,** *177* (1), 876-880.
2. Esmat, M.; Farghali, A. A.; Khedr, M. H.; El-Sherbiny, I. M., Alginate-based nanocomposites for efficient removal of heavy metal ions. *International Journal of Biological Macromolecules* **2017,** *102*, 272-283.

3. Baba, Y.; Masaaki, K.; Kawano, Y., Synthesis of a chitosan derivative recognizing planar metal ion and its selective adsorption equilibria of copper(II) over iron(III)1. *Reactive and Functional Polymers* **1998,** *36* (2), 167-172.
4. Limsuwan, Y.; Rattanawongwiboon, T.; Lertsarawut, P.; Hemvichian, K.; Pongprayoon, T., Adsorption of Cu(II) ions from aqueous solution using PE/PP non-woven fabric grafted with poly(bis[2-(methacryloyloxy) ethyl] phosphate). *Journal of Environmental Chemical Engineering* **2021,** *9* (6), 106440.
5. Lin, X.; Shen, T.; Li, M.; Shaoyu, J.; Zhuang, W.; Li, M.; Xu, H.; Zhu, C.; Ying, H.; Ouyang, P., Synthesis, characterization, and utilization of poly-amino acid-functionalized lignin for efficient and selective removal of lead ion from aqueous solution. *Journal of Cleaner Production* **2022,** *347*, 131219.
6. Mzinyane, N. N.; Ofomaja, A. E.; Naidoo, E. B., Synthesis of poly (hydroxamic acid) ligand for removal of Cu (II) and Fe (II) ions in a single component aqueous solution. *South African Journal of Chemical Engineering* **2021,** *35*, 137-152.
7. Ben Ali, M.; Wang, F.; Boukherroub, R.; Xia, M., High performance of phytic acid-functionalized spherical poly-phenylglycine particles for removal of heavy metal ions. *Applied Surface Science* **2020,** *518*, 146206.
